# High‐Gain PMMA‐Modified Graphene Photodetectors for Dual‐Wavelength Secure Communication Utilizing Distinct Temporal Photoresponses

**DOI:** 10.1002/advs.202501294

**Published:** 2025-05-11

**Authors:** Jinhua Wu, Hao Sun, Ruiling Zhang, Shipeng Yao, Zhangyu Hou, Cun‐Zheng Ning

**Affiliations:** ^1^ Department of Electronic Engineering Tsinghua University Beijing 100084 China; ^2^ College of Integrated Circuits and Optoelectronic Chips Shenzhen Tech. University Shenzhen 518118 China; ^3^ Frontier Science Center for Quantum Information Beijing 100084 China

**Keywords:** graphene, oppositive polar responses, photodetector, secure communication

## Abstract

Photodetectors exhibiting oppositive polar responses at two different wavelengths have been used extensively for secure optical communication. However, current technologies are susceptible to signal cancellation and cross‐talk, due to the time‐independent responses of equal strengths. In this study, a different encryption system based on a PMMA‐modified graphene (PMG) photodetector is demonstrated that shows remarkable wavelength‐dependent temporal responses: the PMG detector shows a largely constant response for wavelengths longer than 580 nm, while a linear time dependence for shorter wavelengths. It is found that the qualitatively different temporal responses are attributed to the photogating effects and the PMMA‐mediated oxygen desorption process, respectively. Moreover, the PMG device achieves exceptionally large normalized‐gain values of 2.18 × 10^−3^ m^2^V^−1^ and 2.97 × 10^−‍5^ ‍m^2^V^−‍1^ for the two cases, respectively, both surpassing previously reported values by an order of magnitude for the bipolar responsive photodetectors in ambient conditions. By harnessing these distinct temporal photoresponses as encrypted and key signals at different wavelengths, an effective encryption system for optical communication is demonstrated that enhances signal recovery and avoids signal cancellation. The research introduces a straightforward yet efficient structural design for photodetectors with temporally distinct photoresponses at specific wavelengths and represents a significant advancement in the field of secure communication.

## Graphical abstract

The bipolar photoresponses with temporally distinguishable characteristics in a PMMA‐modified graphene photodetector are successfully achieved. Through systematic harnessing of these wavelength‐dependent temporal dynamics as encrypted and key signals, an effective encryption system for optical communication systems is developed. This approach demonstrates significant improvement in signal recovery fidelity while effectively preventing signal cancellation between communication channels.

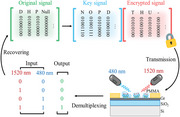

## 1 Introduction

The advancement of secure wireless optical communication systems is crucial to mitigate data breaches during free‐space light transmission.^[^ ^1^, ^2^, ^3^ ^]^ Challenges such as scattering and refraction of signal light in air or water environments over extended distances expose these systems to security vulnerabilities, including potential breaches or unauthorized eavesdropping.^[^ ^4^, ^5^ ^]^ Various strategies have emerged to enhance the security of these systems, including encryption algorithms such as computational temporal ghost imaging^[^ ^6^ ^]^ and multi‐layer chaotic encryption^[^ ^7^ ^]^ Although these methods effectively obscure transmitted data within forged or disordered messages, they fundamentally rely on the secure exchange of encryption keys between transmitters and receivers, leading to challenges involving key distribution capacity, operational speed, and computational resource demands^[^ ^8^ ^]^ Quantum key distribution has emerged as a robust alternative, allowing secure message transmission via unconditionally secure quantum channels.^[^ ^9^, ^10^ ^]^ However, inherent properties of quantum mechanics shorten transmission distances^[^ ^11^ ^]^ and increase system complexity and costs^[^ ^11^ ^]^

Recent advances in photodetector technology show the potential for unique devices capable of differentiating wavelengths to transform cryptographic applications.^[^ ^3^, ^12^, ^13^ ^]^ These wavelength‐distinguished photodetectors allow for the encryption of signals at specific wavelengths while reserving alternative wavelengths for key signaling. Some designs rely upon strategically stacking photosensitive layers or modifying compositions to regulate response wavelengths by manipulating charge injection direction ^[^ ^13^, ^14^, ^15^, ^16^ ^]^ or incident light pathways.^[^ ^2^, ^17^ ^]^ However, these photodetectors frequently complicate operational dynamics within cryptographic systems, necessitating bias adjustments^[^ ^14^ ^]^ specific incident light orientations^[^ ^17^ ^]^ or supplementary camouflaged signal channels^[^ ^2^ ^]^ In contrast, bipolar responsive photodetectors hold significant promise by incorporating positive photoresponse (PPR) or negative photoresponse (NPR) based on illumination wavelengths.^[^ ^1^, ^18^, ^19^, ^20^, ^21^, ^22^, ^23^, ^24^ ^]^ This property allows for differentiation wavelengths with the photocurrent being higher or lower than the dark current, respectively. Numerous advancements have employed diverse strategies to realize bipolar responses, including photoelectrochemical effects,^[^ ^20^, ^21^, ^23^ ^]^ plasmonic effects^[^ ^25^ ^]^ bolometric effect^[^ ^26^ ^]^ photothermoelectric effect^[^ ^27^ ^]^ surface defects,^[^ ^19^, ^28^ ^]^ and gate‐tuning heterostructures.^[^ ^18^, ^29^ ^]^ However, existing P‐N junction devices when operated in photovoltaic modes ^[^ ^1^, ^12^, ^20^, ^25^, ^27^, ^30^, ^31^, ^32^, ^33^ ^]^ typically manifest limited responsivity levels, generally on the order of tens of mAW^−1^.

In contrast, photodetectors utilizing 2D materials,^[^ ^3^, ^18^ ^]^ such as graphene, offer higher photoconductive gain and responsivity due to photogating effects^[^ ^34^ ^]^ Graphene's intrinsic properties, including zero bandgap nature and exceptional mobility, facilitate the development of highly responsive photodetectors with bipolar photoresponses.^[^ ^3^, ^22^, ^35^, ^36^ ^]^ While graphene detectors with complex multi‐layer structures can enhance performance, they also introduce significant fabrication challenges^[^ ^30^, ^37^ ^]^ or heterogeneous integration on a specific substrate.^[^ ^3^ ^]^ Moreover, existing secure optical communication strategies utilizing these advanced photodectors^[^ ^1^, ^3^, ^27^, ^38^ ^]^ encounter challenges such as signal cancellation and cross‐talk resulting from symmetrical time‐dependent photoresponses. Therefore, the exploration of bipolar responses characterized by high responsivity, uncomplicated device structures, and minimal signal cross‐talk is imperative to enhance secure optical communication systems.

To address these challenges, we propose an encryption strategy based on temporal demultiplexing that exploits the distinct time‐domain photoresponses observed in a PMMA‐modified graphene (PMG) photodetector. Our design reveals bipolar asymmetrical and distinct time‐dependent behaviors. The NPR, attributed to oxygen desorption facilitated by PMMA, exhibits a linear decrease when illuminated with wavelengths from 405 nm to 580 nm, reaching peak responsivity of 5.8 × 10^5^ AW^−1^ at 405 nm. In contrast, the PPR maintains an almost time‐independent constant value across a broader range (405 nm to 1650 nm), facilitated by photogating effects enabling maximum responsivity exceeding 1 × 10^4^ AW^−1^ for PPR. In comparative analysis,^[^ ^39^ ^]^ our device achieves remarkable normalized gain values of 2.18 × 10^−3^ m^2^V^−1^ for NPR and 2.97 × 10^−5^ m^2^V^−1^ for PPR. These values outperform prior bipolar‐responsive photodetectors in ambient environments by an order of magnitude. By leveraging the distinct temporal characteristics of photoresponses, we successfully demonstrate a reliable secure optical communication system employing near‐infrared and visible light as encryption signals and keys, respectively. This distinct temporal response promotes multi‐channel encryption and leads to substantial enhancement in signal recovery as compared to existing strategies. Our PMG photodetector presents a straightforward yet highly effective structure, showcasing record‐breaking gain performance. The approach of temporal demultiplexing encryption holds promise for enhancing the reliability of secure optical communication systems.

## 2 Results and Discussion

**Figure 1:**
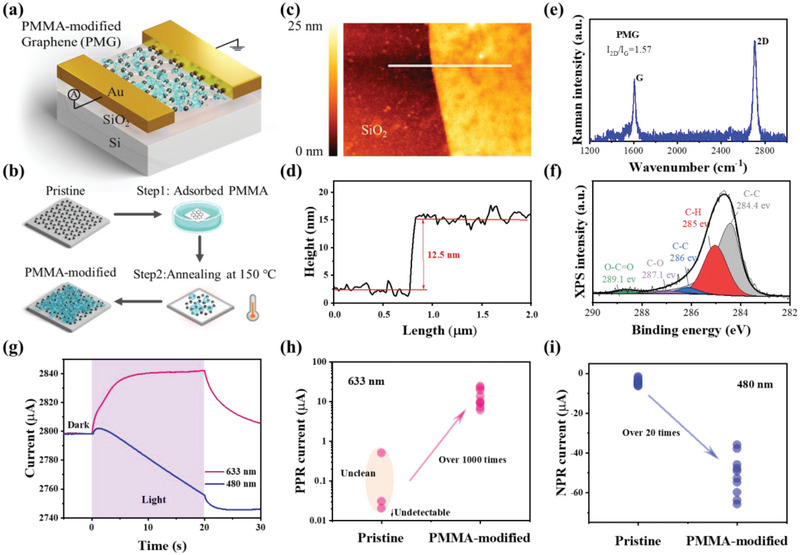
a) Structural diagram of the PMG detector. b) Fabrication processes of PMG. c) AFM image of PMG on a SiO_2_/Si substrate. d) Height profiles along the white line in the AFM image. e) Raman spectrum of PMG. f) XPS spectrum of PMG. The dominant gray peak at a binding energy of 284.4 eV corresponds to the sp2 component of C─C bonding in graphene and is fitted using the asymmetric Doniach‐Sunjic line shape. The red, blue, purple, and green peaks represent different carbon bond states in PMMA: C─H at 285.0 eV, C─C at 286.0 eV, C─O at 287.1 eV, and O─C═O at 289.1 eV, respectively. These peaks are fitted using the Gaussian‐Lorentzian product formula. During peak fitting, background spectra were subtracted using the Shirley algorithm.^[^ ^40^ ^]^ g) I‐t current curves at a source‐drain voltage of 1 V under illumination at 480 nm (2.1 nW) and 633 nm (20.4 nW). h) and f) Comparative analysis of response currents for PPR at 633 nm and NPR at 480 nm across 10 devices with pristine graphene versus PMG.

The device structure depicted in **Figure** **1**a consists of a PMMA‐modified graphene (PMG) layer fabricated on a SiO_2_/Si substrate, with gold (Au) electrodes serving as source‐drain terminals. Planar scanning electron microscope (SEM) imaging (Figure S1, Supporting Information) demonstrates the structural integrity of the graphene‐PMMA heterostructure, with no detectable interfacial defects or layer discontinuities observed at the micrometer scale. The PMG fabrication process is illustrated in Figure 1b. Pristine graphene is synthesized via chemical vapor deposition and subsequently transferred onto the SiO_2_/Si substrate via a wet‐transferring method. Following treatment with a solution of acetone and dissolved PMMA, a thin layer of PMMA is retained on the graphene surface. A 100 nm‐thick layer of gold is then thermally evaporated onto the graphene, defining a channel length of 35 µm. To enhance the adhesion of metal contacts and modify the bonding state between PMMA and graphene, the completed devices undergo annealing at 150 °C for 2 h in a nitrogen atmosphere.

Figure 1c displays the atomic force microscopy (AFM) image of the PMG, with the white line indicating the path along which the height profile is obtained (shown in Figure 1d), revealing a residual PMMA thickness of ≈12 nm. In comparison, AFM thickness profiles (Figure S2, Supporting Information) confirm minimal residue in pristine and PMMA‐removed graphene (<3 nm), contrasting sharply with PMMA‐modified graphene (≈12 nm). The typical Raman spectrum of PMG, shown in Figure 1e, indicates an intensity ratio (I_2D_/I_G_) of the 2D peak and G peak of ≈1.57. A comparison with the Raman spectrum of pristine graphene (Figure S3b, Supporting Information) shows a reduced intensity ratio (I_2D_/I_G_) in the PMG, which is indicative of an enhanced doping effect due to the presence of PMMA. The presence of the 2D peak with a Lorentzian line shape confirms the monolayer nature of the graphene film, while the absence of the defect peak (D peak) at ≈1350 cm^−1^ indicates high‐quality graphene with minimal defects. The X‐ray photoelectron spectroscopy (XPS) spectra in Figure 1f and Figure S4 (Supporting Information) further confirm the presence of residual PMMA in the Carbon (C) 1s core‐level spectra.

Electrical measurements were conducted in ambient environments using a semiconductor parameter analyzer. The representative steady‐state photocurrent versus time (I‐t) curves under illumination at 480 nm and 633 nm are shown in Figure 1g, with a source‐drain voltage of 1 V. Under 480 nm illumination, the currents increase rapidly and then decline almost linearly after the light is turned off. In contrast, illumination at 633 nm results in a positive response current that exceeds dark current levels, producing a nearly flat I‐t curve characterized by a constant photocurrent. The choice of these specific wavelengths illustrates the two distinct response types inherent to the PMG device. Figure 1h,i demonstrate the device performance across ten devices utilizing pristine graphene versus PMG, evidencing a significant enhancement in positive current for PMG devices, with values exceeding three orders of magnitude, while several devices comprising pristine graphene exhibit insufficient current for detection within the same measurement context. The negative current also shows a notable enhancement by an order of magnitude in PMG devices, as depicted in Figure 1i. To validate this enhancement attributable to PMMA, control experiments were performed to remove PMMA from the devices by subjecting them to 370 °C for 1 h in an argon (Ar) atmosphere (10 sccm, 200 Pa). The resultant Raman spectra (Figure S3c, Supporting Information) exhibited broad fluorescent signals from 1200 to 1650 cm^−1^, indicating the presence of amorphous carbon resulting from PMMA fragmentation. Following PMMA removal, photoresponse currents decreased by over one order of magnitude (Figure S5, Supporting Information), underscoring the critical role of PMMA in optoelectronic modulation. These control experiments clearly indicate the critical role of PMMA in facilitating wavelength‐specific bipolar photoresponses.

**Figure 2:**
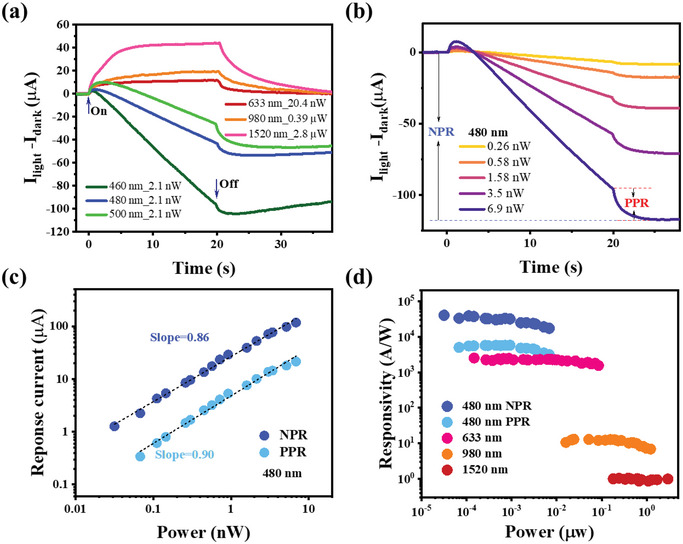
a) Response current curves as a function of time under various illumination wavelengths. The current responses at 980 nm and 1520 nm are multiplied by six, respectively. b) Time‐dependent current curves under varying illumination intensities at 480 nm. c) Power‐dependent current values of PPR and NPR components under 480 nm illumination. d) Power‐dependent responsivity at 480 nm, 633 nm, 980 nm, and 1520 nm.

Further measurements of photoresponse currents across various illumination wavelengths are shown in **Figure** **2**a. The photocurrents at 460 nm, 480 nm, and 500 nm under identical illumination power decrease linearly over time, with the decreasing rate diminishing as the wavelength increases. Conversely, under illumination at 633 nm (20.4 nW), 980 nm (390 nW), and 1520 nm (2.8 µW), the photocurrents display nearly time‐independent behavior, indicating only the presence of the PPR component. Figure 2b represents the 480 nm photoresponse currents at varying illumination powers from 0.26 to 6.9 nW. As the light intensity increases, the peak current value associated with the PPR components initially rises above the dark current, while the NPR component declines at a more pronounced rate, resulting in lower negative values. The PPR component maintains a nearly time‐independent response upon light activation, whereas the NPR component exhibits a steady decay over time. After the light is turned off, the persistent negative photocurrent offset can be attributed to the gradual re‐adsorption of ambient gas molecules on the graphene surface, a phenomenon that exhibits strong dependence on both environmental conditions and the density of surface defects. Complete recovery to the initial conductivity state necessitates prolonged equilibration under controlled conditions.

The two components, PPR and NPR, are separated and plotted in Figure 2c, revealing nearly linear relationships with varying power intensities from 0.03 to 6.9 nW, with slopes of 0.90 and 0.86, respectively. Responsivity (R) is further assessed as a function of illumination intensity at 480 nm, 633 nm, 980 nm, and 1520 nm in Figure 2d, defined as R = |I_light_‐I_dark_|/P, where I_light_, I_dark_, and P represent the response current, dark current, and effective illumination power, respectively. The responsivity remains almost stable across the measured power range, indicating a linear response of almost three orders of magnitude in intensity. We characterized the external quantum efficiency (EQE) across spectral and power domains (Figure S6, Supporting Information), employing the standard relation EQE = |I_light_‐I_dark_|hc/Peλ, where h is Planck's constant, c is the speed of light, e is the electron charge, and λ is the illumination wavelength. The device demonstrates remarkable EQE values exceeding 100% at wavelengths below 980 nm, attributable to PMMA‐induced charge multiplication effects. This gain mechanism maintains exceptional performance in the near‐infrared range, sustaining EQE > 80% at 1520 nm. As shown in Figure S7 (Supporting Information), the linear dynamic range (LDR) exceeds 40 dB at 480 nm and reaches 55 dB at 633 nm, demonstrating broadband operation capability.

**Figure 3:**
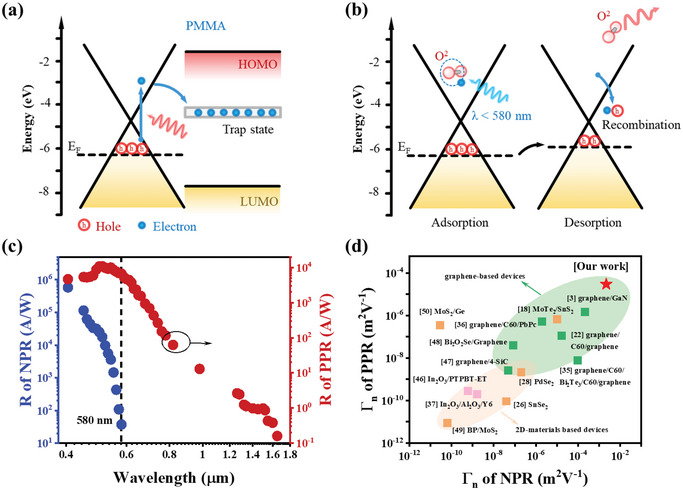
Energy band diagram explanation of the generation mechanisms for PPR a) and NPR b) under different illumination conditions. c) Wavelength‐dependent responsivity of PPR and NPR. d) Reported normalized gain values (Γ_n_) for PPR and NPR compared to our device.

To elucidate the underlying mechanisms responsible for the distinct temporal characteristics, energy band diagrams of PMG are illustrated in **Figure** **3**a,b. As depicted in Figure 3a, the polar ester groups of PMMA generate a localized trap state near – 4.2 eV, with a distribution width of ≈0.4 eV.^[^ ^41^, ^42^ ^]^ When photogenerated electron carriers in graphene exceed this trap state energy, electrons transfer to PMMA. The resultant photogating effect of the trapped electron carriers in PMMA induces an increase in the conductivity of the p‐type graphene, leading to a more prominent PPR across the wavelength range of 405 nm to 1650 nm compared to pristine graphene. The photogating effect is further confirmed through gate‐dependent transition measurements, as demonstrated in Figure S8 (Supporting Information). Concurrently, the presence of PMMA enhances the adsorption of air molecules, including oxygen,^[^ ^40^ ^]^ which captures electrons and forms negatively charged ions,^[^ ^43^, ^44^ ^]^ as shown in Figure 3b. When exposed to light with sufficient energy to surpass the binding energy between air molecules and graphene, desorption occurs,^[^ ^45^ ^]^ enhancing the electron‐hole recombination and leading to reduced hole photoconductivity and negative current. A sustained illumination consequently leads to a continuous current decrease at a specific rate. Our device exhibits a pronounced NPR in the wavelength range from 405 nm to 580 nm, with the lower limit determined by the capabilities of our measurement instruments.

To further investigate the wavelength‐specific response properties, we measured the responsivity of the PMG device under illumination with a supercontinuum light source equipped with a monochromator. For short‐wavelength illumination below 580 nm, we calculated the responsivity separately for the NPR and PPR components. Figure 3c shows that the PPR spans a broad wavelength range from 405 to 1650 nm, achieving a maximum responsivity exceeding 1 × 10^4^ AW^−1^, significantly surpassing that of pristine graphene. Systematic comparisons of wavelength‐dependent responsivity and response kinetics between PMMA‐modified and untreated graphene (Figure S9, Supporting Information) confirm enhanced photoresponse magnitudes and prolonged carrier lifetimes induced by photogating effects. While our experimental measurements demonstrate reliable photodetection up to 1.5 µm, the fundamental operating mechanism suggests a potential response extending to 3.0 µm. This theoretical limit originates from the 0.4 eV interfacial barrier between graphene's work function (−4.6 eV) and PMMA's trap states (−4.2 eV), corresponding to the energy of 3.0 µm photons (E = hc/λ).

Conversely, the NPR occurs solely under illumination below 580 nm, with a peak responsivity exceeding 5.8 × 10^5^ AW^−1^ at 405 nm. As wavelength increases, the responsivity of NPR declines sharply until it cuts off at 580 nm. The detectivity (D*), defined as D* = R/(2qI_dark_/S)^1/2^, where q is the electron charge and S is the effective illuminated area, is calculated in Figure S10 (Supporting Information). The highest detectivity values of NPR and PPR are 1.05 × 10^14^ and 1.97 × 10^12^ Jones, respectively. The noise equivalent power (NEP) in Figure S11 (Supporting Information) was also determined by dividing the noise spectral density by the responsivity, yielding an ultra‐low value of 1.38 × 10^−14 ^W Hz^−1/2^ at 405 nm. The performance enhancement stems from PMMA‐mediated interface engineering, which simultaneously improves responsivity and noise suppression. The achieved NEP of 10^−‍14^ W Hz^−1/2^ competes with state‐of‐the‐art photodetectors, while maintaining high LDR for practical applications.

Another important intrinsic parameter related to material properties is the normalized gain Γ_n_, which incorporates channel length and source‐drain voltage. ^[^ ^39^ ^]^ It is calculated using the formula: Γ_n_ = RL^2^ω/ηqV, with R, L, ω, V, and η denoting responsivity, channel length, photon energy, source‐drain voltage, and quantum efficiency (assuming to be 1 for simplicity), respectively. The maximum normalized gain values of NPR and PPR are 2.18 × 10^−3^ m^2^V^−1^ and 2.97 × 10^−5^ m^2^V^−1^, respectively, as shown in Figure 3d. These values exceed previously reported bipolar‐response photodetectors operating in ambient environments by an order of magnitude.^[^ ^3^, ^18^, ^22^, ^26^, ^28^, ^35^, ^36^, ^37^, ^46^, ^47^, ^48^, ^49^, ^50^ ^]^ Detailed parameters are provided in Table S1 (Supporting Information). The responsivities of various devices from the same processing batch and different batches are displayed in Figure S12 (Supporting Information), showing histograms of the responsivity at 480 nm excitation for NPR and 633 nm and 1520 nm for PPR, highlighting stable wavelength‐specific bipolar photoresponses with consistent responsivity levels across all 18 devices. Long‐term response stability was also assessed for devices stored in a nitrogen atmosphere, revealing consistent responsivity for both PPR and NPR over a period of 300 days, as shown in Figure S13 (Supporting Information).

**Figure 4:**
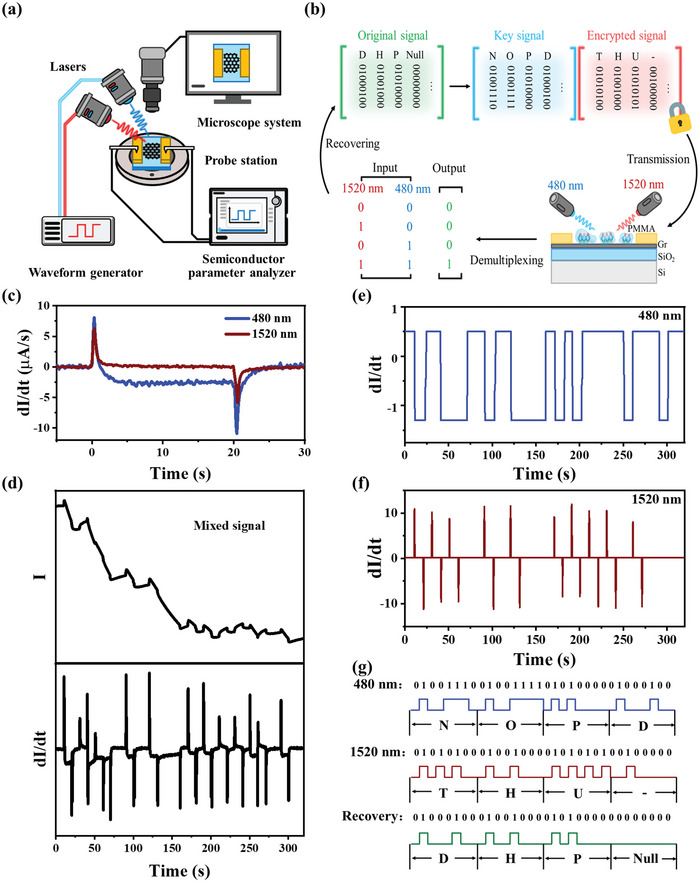
a) Schematic of the secure optical communication system. b) Illustration of secure encoding and encryption processes in optical communication systems, where the PMG photodetector is illuminated with 1520 nm and 480 nm light as encrypted and key signals, respectively. c) Current change rate (dI/dt) versus time (t) under 480 nm and 1520 nm illumination. d) I‐t curve of the original mixed signal and the value after differential operation. e) Demultiplexed signal from 480 nm channel. f) Demultiplexed signal from 1520 nm channel. g) Recovered encryption information from the two channels.

Building on the distinct temporal photoresponses observed at specific wavelengths, we propose a secure encoding encryption system for optical communication. The experimental setup, illustrated in **Figure** **4**a, consists of three main components: (1) a probe station with integrated microscopy for real‐time monitoring of probe‐electrode contacts, (2) a dual‐channel waveform generator controlling laser illumination (with Channel 1 generating encrypted optical codes and Channel 2 providing decryption keys), and (3) a semiconductor parameter analyzer for electrical characterization. This configuration enables simultaneous optical excitation and electrical readout while ensuring precise alignment verification through microscopic observation. The encrypted optical input and synchronized key signal facilitate secure photodetection measurements, with all electrical responses recorded through the parameter analyzer under controlled environmental conditions. To evaluate the effectiveness of our encryption system, we conducted experiments to encrypt corresponding light sources at wavelengths of 480 nm and 1520 nm. As illustrated in Figure 4b, the original signal is first converted into two separate channels following predefined encryption rules: the encrypted signal and the corresponding key signal. This framework ensures the security of the actual information, even if the light source is intercepted during transmission. The receiver detects the mixed signals based on the time‐dependent responses of the photodetector at different wavelengths, characterized by a time‐independent constant value for one signal and a linearly decreasing response for the other, enabling demultiplexing through signal processing. Finally, the decryption rules are employed to recover the actual signal.

As shown in Figure 4c, the differential operation on I‐t curves reveals fundamentally distinct behaviors: 480 nm illumination produces a steadily negative current change rate (≈2.7 µA s^−1^) while 1520 nm illumination maintains a near‐zero rate (≈0.03 µA s^−1^). We have now quantified these temporal characteristics through dedicated measurements, determining a decay time constant of 19 s for 480 nm and establishing the stability at 1520 nm. The absolute ratio of current change rates between these wavelengths exceeds 10:1 after 2.5 s (Figure S14, Supporting Information), creating a robust time‐domain signature for encryption. This differential temporal response has been intentionally engineered to serve as an additional security parameter in optical communication systems, where the distinct wavelength‐dependent time signatures function as physical‐layer encryption keys. The distinct temporal attributes of NPR and PPR contribute to improved signal recovery and mitigate cancellation and signal cross‐talk, addressing the limitations observed in previous studies with symmetrical photoresponses.^[^ ^3^, ^27^, ^30^ ^]^ Additionally, considering the high absorption coefficient of near‐infrared light in seawater, we demonstrated an encryption system utilizing visible light, with 633 nm and 480 nm illumination acting as the encrypted and key signals, respectively (see Figure S15, Supporting Information).

In this context, “0′ indicates the light is off, and ‘1′ indicates the light is on. The signal in the 1520 nm channel was encrypted using ASCII codes ‘T”, “H”, “U”, and “‐”, while the key signal in the 480 nm channel transmitted the ASCII codes “N”, “O”, “P”, and “D”. By applying the decryption rules based on the truth table depicted in Figure 4b, we successfully recovered the actual signal, with simultaneous signal values of “1′ in both channels indicating a ‘1′ state. Figure 4d shows the original mixed signal detected in the I‐t current curve alongside the corresponding dI/dt curve after the differential operation. In the dI/dt curve, the NPR exhibits a stable negative value. After smoothing the dI/dt signal in Figure 4d, the response for the 480 nm channel is determined by the negative current change rate in Figure 4e. Through the subtraction of the NPR influence from the dI/dt signal in Figure 4d, the subsequent isolation of the PPR state at 1520 nm is displayed in Figure 4f. By leveraging both the temporal characteristics of NPR and PPR, we successfully demultiplexed the two‐channel signals and decrypted the original signal into ASCII codes ‘D”, “H”, “P”, and “Null” (Figure 4g). The response speed in our system is dictated by the slow recombination kinetics of trapped carriers, with the RC constant of the device^[^ ^51^ ^]^ and carrier mobility playing negligible roles due to the photogating‐dominated mechanism. Figure S16 (Supporting Information) presents the frequency response characteristics of the photodetector, revealing a 3 dB bandwidth of 0.6 Hz. This bandwidth corresponds to a maximum achievable data transmission rate of 0.6 bits per second under the current device configuration. The key to optimizing response speed is to reduce the trap state density through improved interface engineering to reduce the carrier lifetimes.

In conclusion, our study demonstrates the feasibility of achieving bipolar photoresponses with distinct temporal characteristics in a PMMA‐modified graphene photodetector, presenting a simpler and more efficient alternative to previous devices without complex fabrication processes. Notably, this device exhibits remarkable normalized gain values of 2.18 × 10^−3^ m^2^V^−1^ and 2.97 × 10^−5^ m^2^V^−1^ for NPR and PPR, respectively. These values surpass previously reported results by an order of magnitude, highlighting the superior performance of our device. Another contribution of our study lies in enhancing the understanding of the unique wavelength‐dependent photoresponsive mechanisms of photodetectors. We elucidate that the bipolar behavior can be attributed to the desorption of oxygen induced by residual PMMA and the photogating effects between PMMA and graphene. In contrast to the photogating effects in most graphene‐based devices cooperating with other semiconductor materials, absorption is dependent on the semiconductor material, leading to a limited spectral response range. In comparison, the PMG detector exhibits a broader response spectrum due to the wider absorption spectrum range of graphene. The PPR in our device can respond to longer or lower wavelengths without experimental constraints. These insights contribute significantly to the underlying fundamental properties governing photodetectors.

Of particular interest are the unique temporal properties observed in our PMG device. The NPR consistently displays a linearly decreasing trend, while the PPR maintains a constant current independent of time. Leveraging these distinctive temporal characteristics, we propose the implementation of a reliable information encryption system in optical communications. By converting the actual signal into two separate channels with encrypted and key signals based on predefined encryption rules, we ensure the security of the actual signal, even if any channel is eavesdropped during message transmission. Enhanced signal recovery of the encrypted signal can only be achieved by demultiplexing of two corresponding channels. Given that each channel possesses distinct asymmetrical temporal characteristics, this recovery approach effectively prevents signal cross‐talk and cancellation. Our experiments demonstrate the encryption capabilities of PMG devices under varying wavelengths in both the near‐infrared and visible bands, showcasing its adaptability and potential in secure optical communications.

While the current device demonstrates promising performance, several potential pathways could further enhance its capabilities. The observed dark current might be suppressed through asymmetric electrode designs that create controlled Schottky barriers, as demonstrated in similar low‐dimensional systems.^[^ ^52^, ^53^ ^]^ The response time could potentially be improved by engineering the PMMA‐graphene interface to reduce trap‐state effects, though this would require careful optimization to maintain the beneficial photogating effects. Alternative device architectures, such as back‐gated configurations^[^ ^54^ ^]^ may offer additional degrees of freedom for performance tuning. Although PMMA establishes the current spectral response characteristics, alternative polymer coatings with tailored electronic structures could potentially extend photodetection to longer wavelengths. By engineering materials with modified polar functional groups, one could systematically tune both trap state energies and densities at the graphene interface. Such modifications would enable control over the interfacial energy barrier, potentially shifting the photogating activation threshold while maintaining the essential security features. Moreover, future iterations could exploit heterojunction architectures or plasmonic field localization to extend secure optical communication into the mid‐infrared regime, as proposed in recent studies.^[^ ^55^, ^56^ ^]^ These potential directions, while beyond the scope of the current study, represent interesting opportunities for future investigations in graphene‐based photodetector development.

Our device holds immense potential for advancing secure optical communications, providing simplicity, effectiveness, and superior performance metrics. The significant improvement in performance metrics opens up new possibilities for developing advanced optoelectronic devices, including optical synaptic devices^[^ ^28^ ^]^ and logic gates.^[^ ^21^, ^30^, ^57^ ^]^ This research not only contributes to the understanding of unique wavelength‐dependent photoresponsive mechanisms but also paves the way for the design and implementation of secure communication technologies in the field of optical communications.

## 3 Experimental Section

### Device Fabrication

Graphene is synthesized by chemical vapor deposition (vendor: six‐carbon technology) and then transferred to a 300 nm SiO_2_ / highly p‐doped Si (0.001‐0.005 Ωcm^−1^) substrate by a wet transfer method^[^ ^58^ ^]^ The fabrication process of PMG involves placing the sample in a petri dish with the addition of 20 µL anisole to dissolve the PMMA at a 3% concentration and 10 mL acetone, and then evaporating the acetone, leaving a residual layer of PMMA on top of graphene. Subsequently, 100 nm Au electrodes with a 35 µm channel length were deposited by thermal evaporation using a G200 transmission electron microscopy copper mesh grid as a mask.

### Device Characterization

Raman spectroscopic data were collected using a Princeton spectrometer with a 514 nm laser as the excitation source. AFM measurements were performed at room temperature using a SNOM microscope, and XPS data were obtained using a scanning XPS microscope (PHI Quantera II). Electrical measurements were conducted in the ambient environment using a semiconductor parameter analyzer (Keysight B2900A) under varying illumination power. The device is illuminated by a supercontinuum laser (Fianium SC450) with a monochromator. The dual‐channel modulated output signal was controlled by a waveform generator (RIGOL DG4602).

## Conflict of Interest

The authors declare no conflict of interest.

## Supporting information



Supporting Information

## Data Availability

The data that support the findings of this study are available from the corresponding author upon reasonable request.
