# Supplementary material for: High‐Gain PMMA‐Modified Graphene Photodetectors for Dual‐Wavelength Secure Communication Utilizing Distinct Temporal Photoresponses
Source: Adv Sci (Weinh). 2025 May 11;12(25):2501294. doi: 10.1002/advs.202501294 (PMC12224954; doi:10.1002/advs.202501294)
Supplement: Supplementary file 1 — Supporting Information [file ADVS-12-2501294-s001.docx]

**Supplementary Information**

**High-gain PMMA-modified Graphene Photodetectors for Dual-wavelength Secure Communication Utilizing Distinct Temporal Photoresponses**

Jinhua Wu^1, 2, 3^, Hao Sun^1, 3, *^, Ruiling Zhang^1, 2, 3^, Shipeng Yao^1, 2, 3^,

Zhangyu Hou^1, 2, 3^, Cun-Zheng Ning^1, 2, 3, *^

^1^Department of Electronic Engineering, Tsinghua University, Beijing, 100084, China

^2^College of Integrated Circuits and Optoelectronic Chips, Shenzhen Tech. University, Shenzhen, 518118, China

^3^Frontier Science Center for Quantum Information, Beijing, 100084, China

*Correspondence to: haosun@mail.tsinghua.edu.cn, ningcunzheng@sztu.edu.cn

[I. SEM image of the photodetector 2](#_Toc197029807)

[II. AFM analysis of graphene surface morphology 3](#_Toc197029808)

[III. Raman characterization of graphene crystallinity 4](#_Toc197029809)

[IV. XPS analysis of graphene chemical composition 5](#_Toc197029810)

[V. Comparison of photoresponses: PMMA-modified vs. PMMA-removed graphene 6](#_Toc197029811)

[VI. Power-dependent external quantum efficiency (EQE) 7](#_Toc197029812)

[VII. Linear dynamic range (LDR) of the photoresponses 8](#_Toc197029813)

[VIII. Transition curve of the photodetector 9](#_Toc197029814)

[IX. Comparison of photoresponses: PMMA-modified vs. pristine graphene 10](#_Toc197029815)

[X. Wavelength-dependent detectivity of PPR and NPR 11](#_Toc197029816)

[XI. Noise-equivalent power (NEP) characterization 12](#_Toc197029817)

[XII. Stability of responsivity at different wavelengths. 13](#_Toc197029818)

[XIII. Long-term stability of photoresponses. 14](#_Toc197029819)

[XIV. Absolute time-dependent ratio of the photocurrent change rate 15](#_Toc197029820)

[XV. Wavelength-selective encryption demonstration (630 nm and 480 nm) 16](#_Toc197029821)

[XVI. Frequency-dependent photocurrents 18](#_Toc197029822)

[XVII. Performance comparison with state-of-the-art photodetectors 19](#_Toc197029823)

[References. 20](#_Toc197029824)

# SEM image of the photodetector


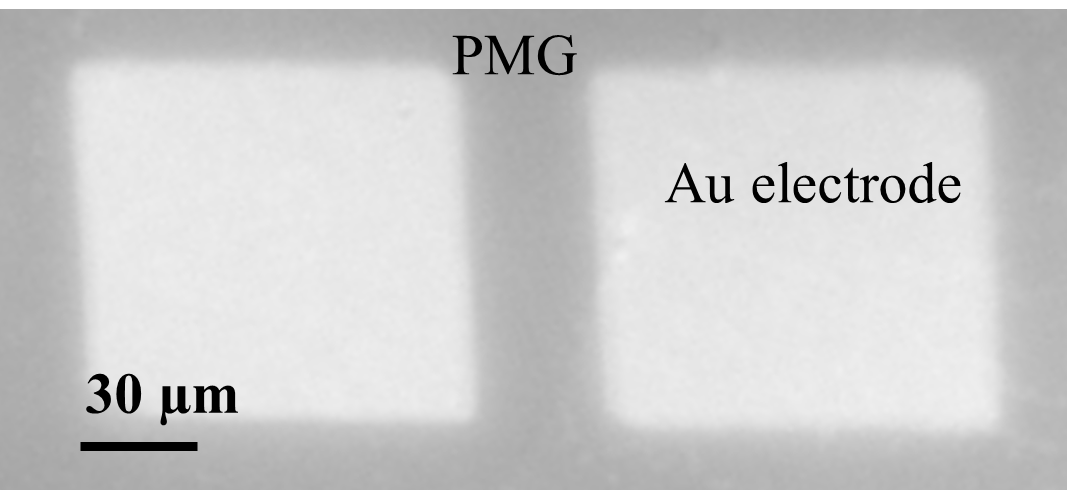


Figure S1. SEM image of the photodetector device

Figure S1 presents the planar scanning electron microscopy (SEM) characterization of the graphene-PMMA heterostructure, showing preserved structural integrity with no detectable interfacial defects or interlayer discontinuities at the micrometer scale.

# AFM analysis of graphene surface morphology


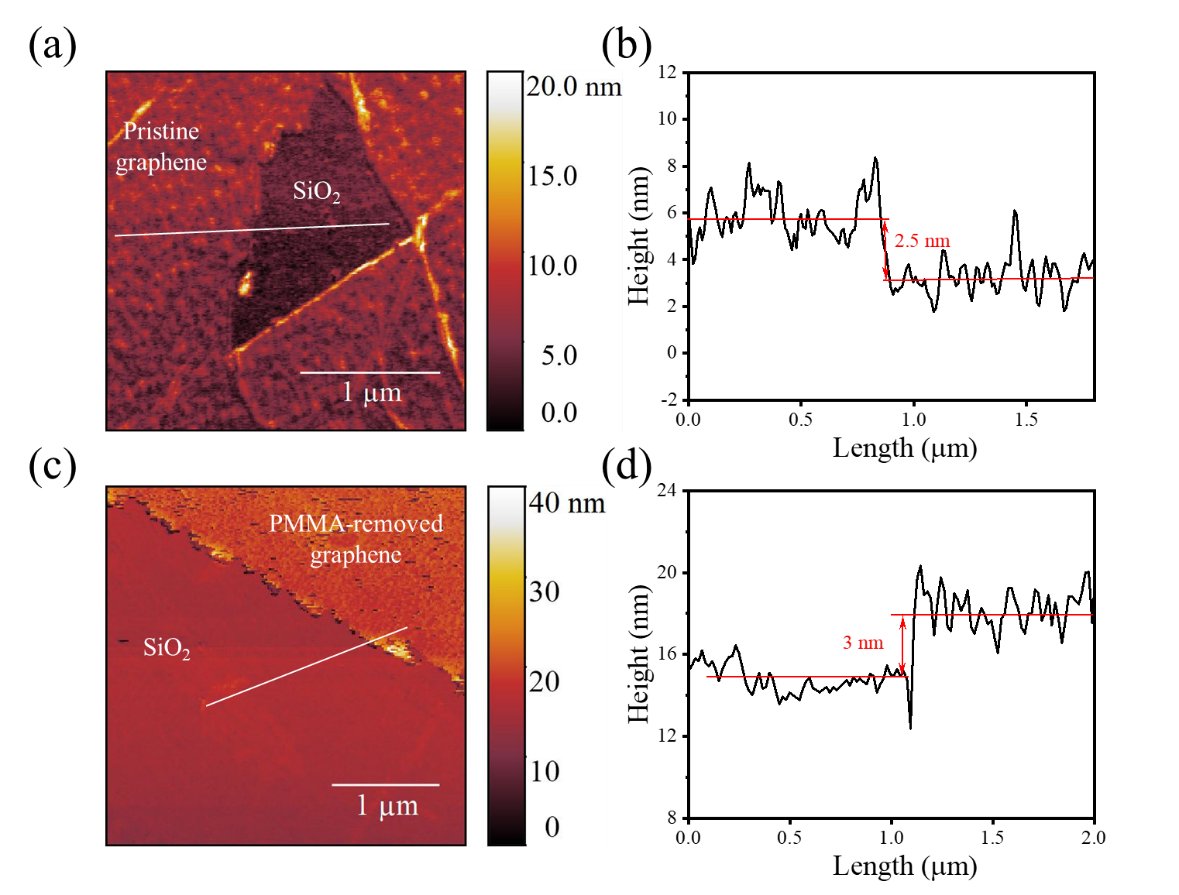


Figure S2. (a) AFM image of untreated graphene on a SiO_2_/Si substrate. (b) Corresponding height profiles along the marked white line in (a), showing a thickness of 2.5 nm. (c) AFM image of PMMA-processed graphene after removal treatment on a SiO_2_/Si substrate. (d) Height profiles along the white line in (c).

Figure S2 presents comparative atomic force microscopy (AFM) analyses of graphene samples. Panel (a) displays the AFM image of untreated graphene on a SiO_2_/Si substrate, with corresponding height profiles along the marked white line shown in (b). The pristine graphene exhibits a characteristic thickness below 2.5 nm, slightly exceeding the theoretical monolayer value (~0.34 nm) due to substrate interactions and instrumental detection limits. Panels (c) and (d) respectively show the AFM image and height profiles of PMMA-removed graphene on an identical substrate. Post-treatment thickness measurements reveal a significant reduction to sub-3 nm levels, demonstrating efficient removal of polymeric residues.

# Raman characterization of graphene crystallinity


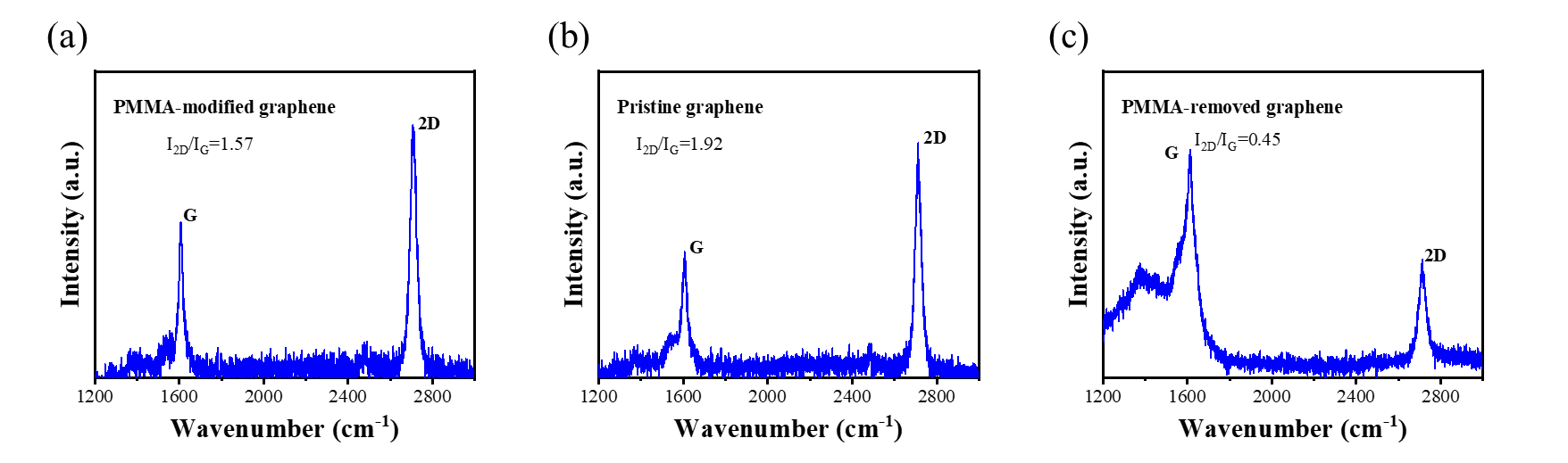


Figure S3. Raman spectra of graphene in various states: (a) PMMA-modified graphene, (b) pristine graphene, (c) PMMA-removed graphene.

Figure S3 shows different Raman spectra of graphene in various states. The intensity ratio of the 2D peak and G peak in PMMA-modified graphene is 1.57 (Fig. S3(a)), which is lower than the value of 1.92 observed in pristine graphene (Fig. S3(b)). This lower intensity ratio reveals that the presence of PMMA enhances the doping effect. To remove the PMMA layer, the PMMA-modified graphene was subjected to heating at 370 ℃ in an argon atmosphere. The Raman spectrum of PMMA-removed graphene (Fig. S3(c)) exhibits a broad fluorescence signal from 1200 to 1650 cm^-1^, indicative of amorphous carbon resulting from PMMA fragmentation.

# XPS analysis of graphene chemical composition


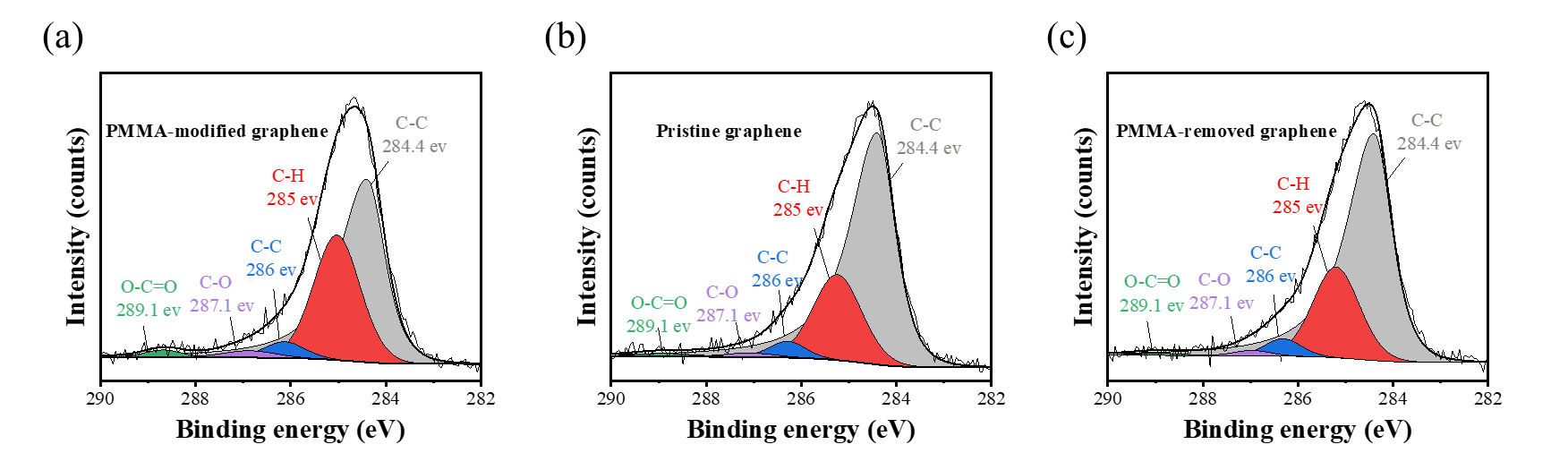


Figure S4. XPS spectrum of graphene in various states: (a) PMMA-modified graphene, (b) pristine graphene, (c) PMMA-removed graphene.

In comparison to the PMMA-modified graphene in Fig. S4(a), the intensity of peaks related to different bonding states in PMMA in Fig. S4(b) and (c) is significantly reduced, suggesting minimal PMMA residue on the graphene. The dominant gray peak at a binding energy of 284.4 eV corresponds to the sp2 component of C-C bonding in graphene. The peaks are color-coded to represent different carbon bonding states in PMMA: C-H at 285.0 eV (red), C-C at 286.0 eV(blue), C-O at 287.1 eV(purple), and O-C=O at 289.1 eV(green).

# Comparison of photoresponses: PMMA-modified vs. PMMA-removed graphene


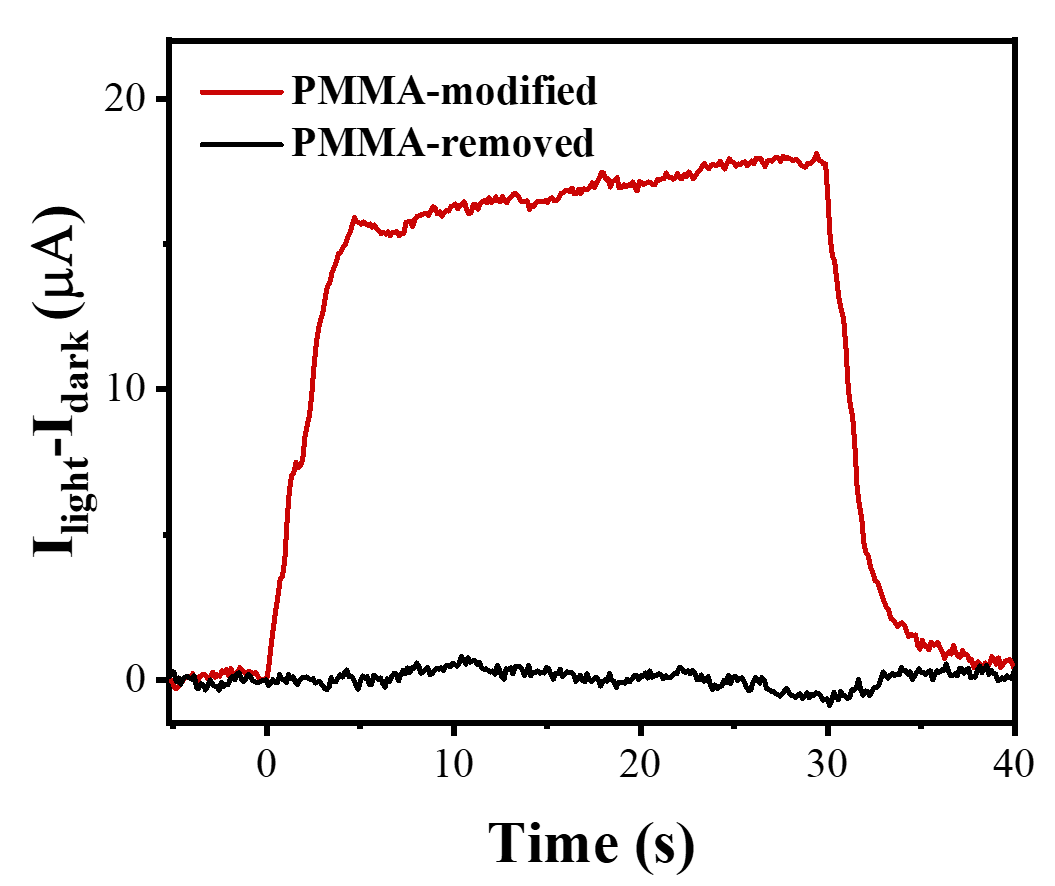


Figure S5. Comparative photoresponse characteristics under 633 nm illumination of PMMA-modified graphene (red) versus PMMA-removed graphene (black) photodetectors at 1V bias.

The photoresponse analysis in Fig. S5 reveals a stark functional contrast between device configurations. PMMA-modified graphene demonstrates robust photocurrent generation. In contrast, PMMA-removed devices exhibit near-baseline signal levels, representing one order of magnitude reduction in photoresponsivity. This dramatic performance degradation correlates with the removal of PMMA-derived charge trapping centers, as evidenced by complementary AFM and XPS characterization.

# Power-dependent external quantum efficiency (EQE)

**
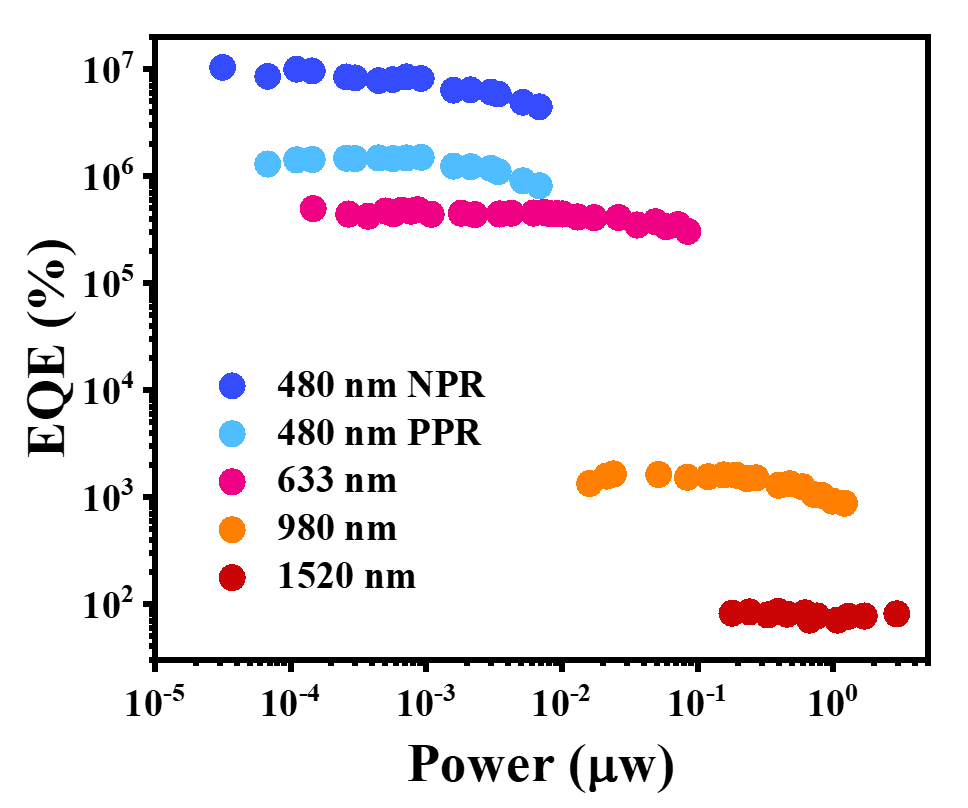
**

Figure S6. Power-dependent EQE under varying wavelengths.

Figure S6 presents power-dependent external quantum efficiency (EQE) analysis under varying wavelengths. EQE At representative wavelengths (480 nm, 633 nm, and 980 nm), power-dependent EQE values exceeding 100 %, attributable to PMMA-induced charge multiplication effects. This gain mechanism maintains exceptional performance in the near-infrared range, sustaining EQE > 80% at 1520 nm.

# Linear dynamic range (LDR) of the photoresponses

The linear dynamic range (LDR), defined as the linear light intensity dependence of the photocurrent, was calculated using the following equation:

$$LDR=20\log(\frac{I_{upper}}{I_{lower}})$$

where *I_upper_* and *I_lower_* are the maximum and minimum limits of the intensity of light. Extracted data from Fig. S7, the PMMA-modified device exhibits an LDR of >40 dB at 480 nm and 55 dB at 633 nm, with a gradual reduction to ∼20 dB at 1520 nm.


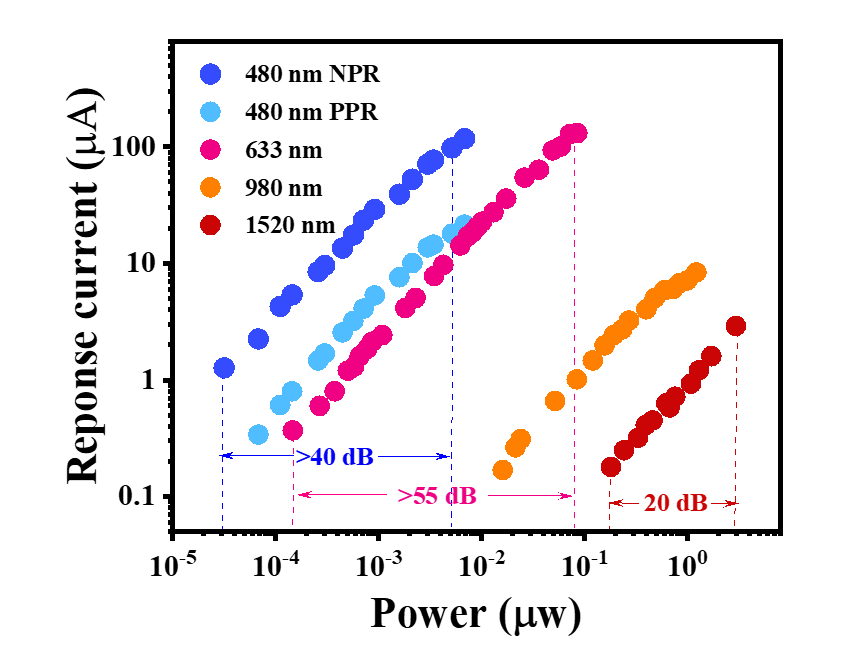


Figure S7. LDR characteristics of the photoresponses at different wavelengths

# Transition curve of the photodetector

*
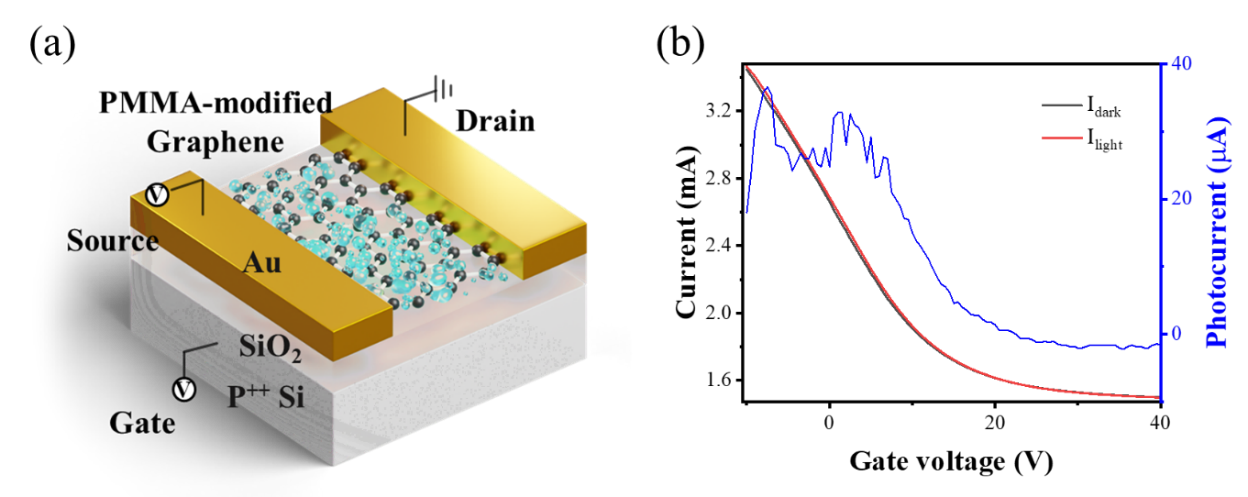
*

Figure S8. (a) Schematic of the gate-tunable device structure. (b) transition curve and photocurrent at different gate voltages.

Figure S8(a) illustrates a schematic of the gate-tunable device structure employing a fixed 1 V source-drain voltage. The transition curve in Fig. S8(b) exhibits characteristic asymmetry and broadening under gate voltage modulation. This behavior is attributed to strong interface doping and Fermi level pinning effects induced by gold-graphene contacts^1^. Compared to the dark condition, the transition curve shifts to the right, which aligns with electron-trapping effects^2^. Notably, the calculated photocurrent in Fig. S8(b) undergoes polarity reversal with gate voltage variation, though maintaining minimal negative values at high gate voltages. These collective observations provide compelling evidence for photogating-dominated operation in our device architecture.

# Comparison of photoresponses: PMMA-modified vs. pristine graphene

*
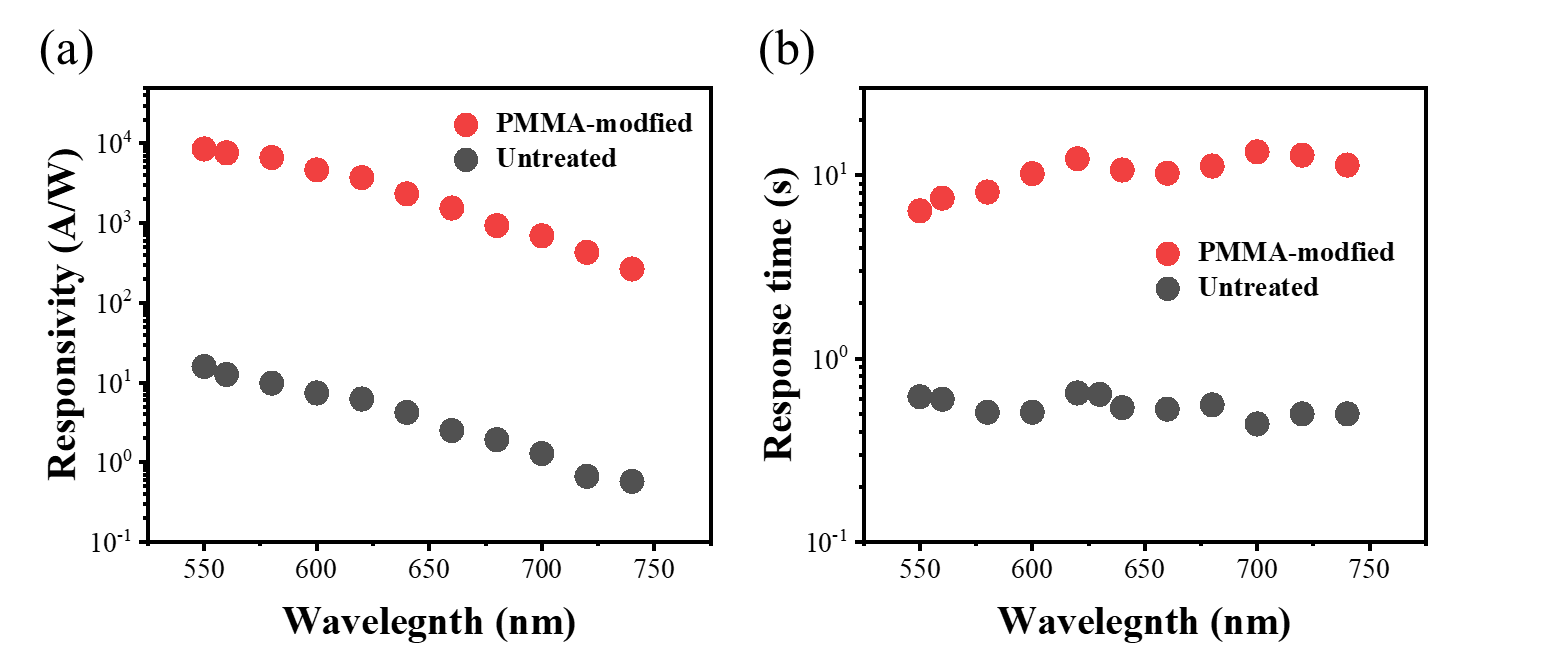
*Figure S9. Comparison of photoresponses between the PMMA-modified graphene and the untreated graphene. (a) Responsivity. (b) Response time.

The PMG devices were fabricated across three independent batches, exhibiting a thickness variation of ~ 2 nm while maintaining rigorously controlled channel lengths of 35 μm. To systematically compare photoresponse characteristics, we performed wavelength-dependent measurements on both PMG and pristine graphene devices. As shown in Fig. S9(a), PMG demonstrates superior responsivity across the tested spectrum, with pristine graphene showing significantly attenuated responses, particularly at longer wavelengths. This performance gap is further quantified in the temporal domain: Fig. S9(b) reveals that PMG exhibits response times prolonged by an order of magnitude relative to pristine graphene. These observations collectively validate the proposed photogating mechanism, where PMMA-induced trap states generate long-lived carriers that simultaneously enhance responsivity through charge multiplication while inherently limiting response speed due to delayed recombination kinetics.

# Wavelength-dependent detectivity of PPR and NPR


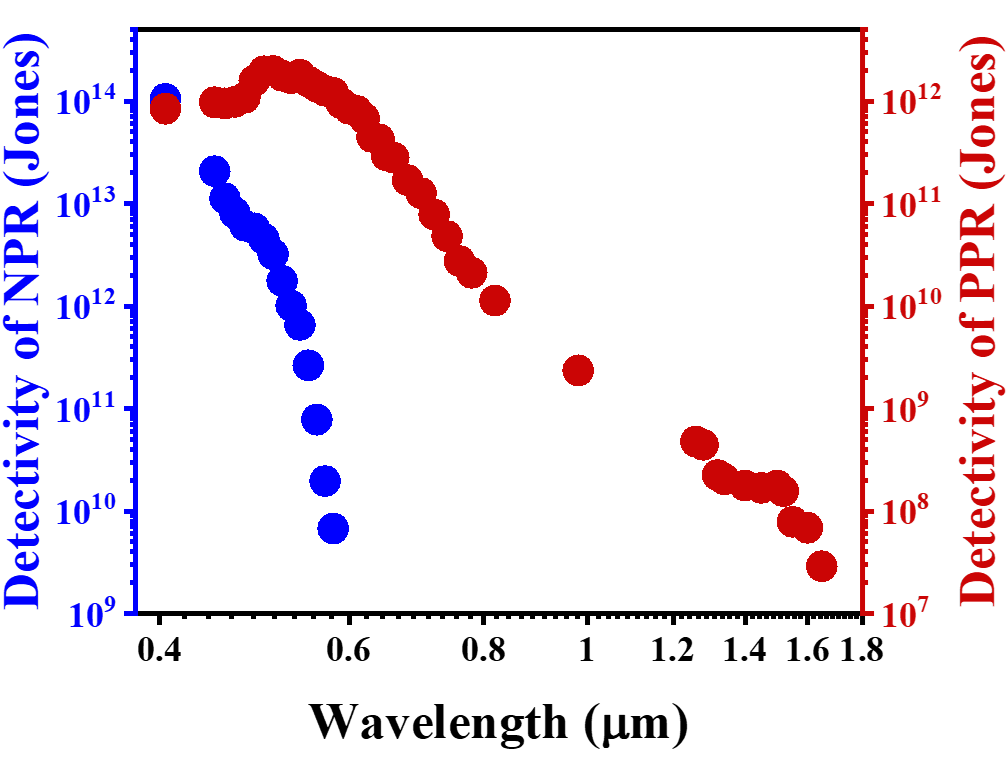


Figure S10. Wavelength-dependent detectivity of PPR and NPR.

# Noise-equivalent power (NEP) characterization

The noise equivalent power (NEP) is defined by the following equation:

$$NEP=\frac{S(f_{n})}{R}$$

where S(*f_n_)* is the noise spectral density and *R* is the responsivity. The noise spectral density is shown in Fig. S11(a), and the calculated NEP values are presented in Fig. S11(b). The device achieves an NEP of of 1.38×10^-14^ at 405 nm.”

*
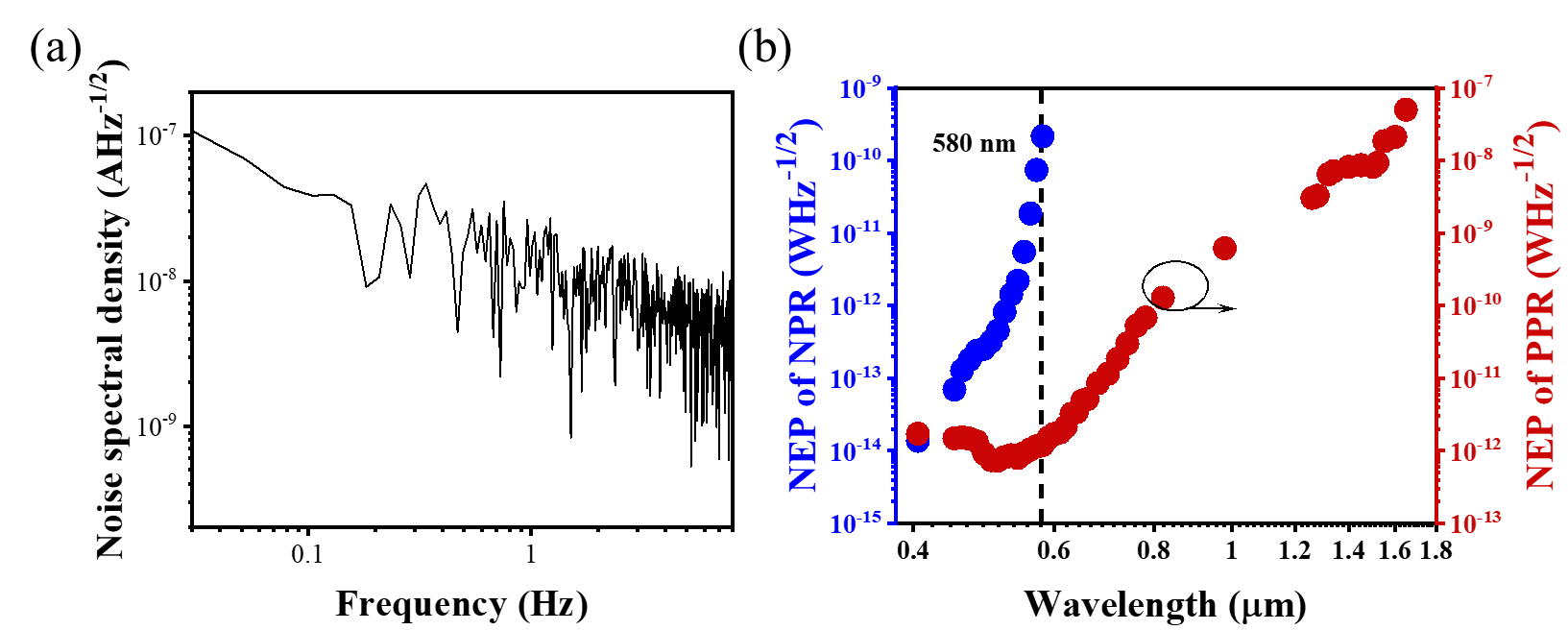
*

Figure S11. (a) Noise spectral density of the device. (b) Wavelength-dependent noise equivalent power (NEP) for both negative photoresponse (blue) and positive photoresponse (red).

# Stability of responsivity at different wavelengths.


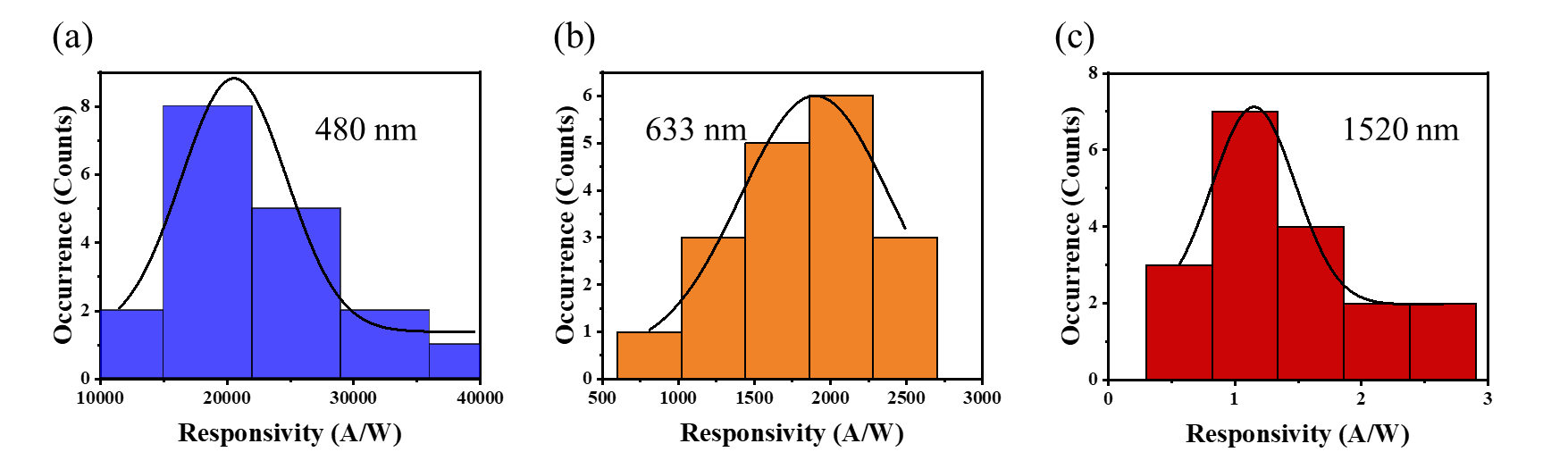


Figure S12. Responsivity measurements of 18 devices at different wavelengths: (a) 480 nm, (b) 633 nm (c) 1520 nm.

We measured the responsivity of 18 devices at different wavelengths, as shown in Fig. S12. The responsivity shows good stability among different devices.

# Long-term stability of photoresponses.


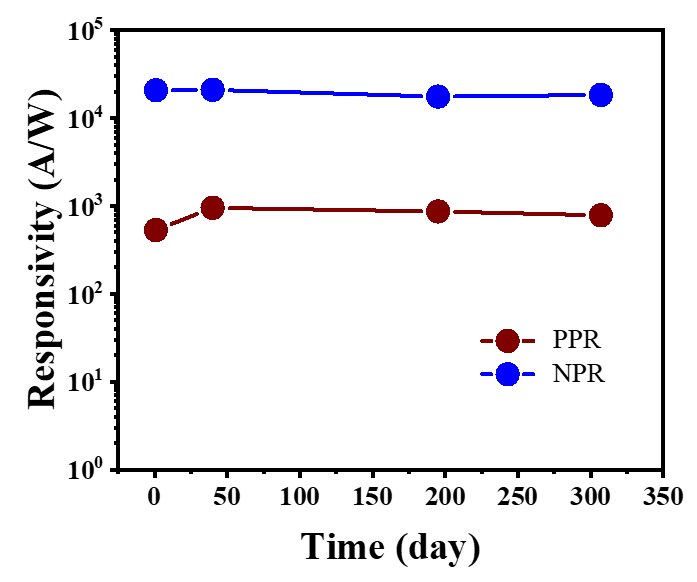


Figure S13. Long-term responsivity stability assessment of the PPR and NPR.

Long-term response stability assessment for devices stored in a nitrogen atmosphere, revealing consistent responsivity for both PPR and NPR over a 300-day period, as shown in Fig. S13. A slight decrease in NPR responsivity can be attributed to reduced oxygen and water adsorption in the environment, while PPR responsivity exhibits an increase, indicating an enhanced photogating effect between PMMA and graphene.

# Absolute time-dependent ratio of the photocurrent change rate


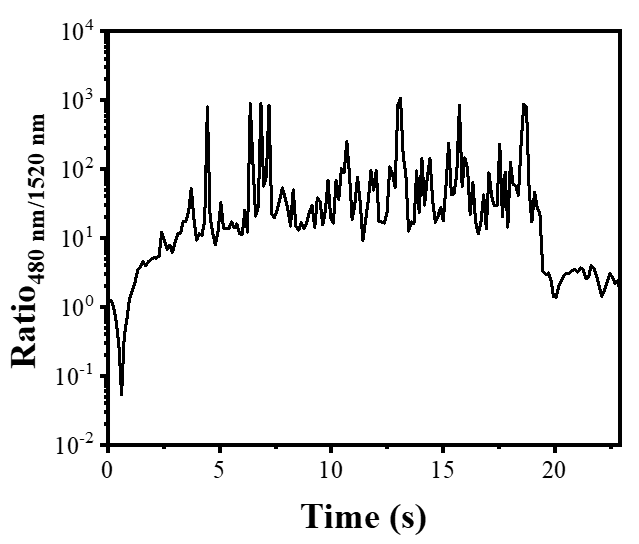


Figure S14. Absolute time-dependent ratio of current change rate under 480 nm and 1520 nm illumination.

Figure S14 quantitatively compares the temporal response characteristics under 480 nm and 1520 nm illumination by plotting the absolute ratio of their current change rates as a function of time. The analysis reveals a striking divergence in photoresponse dynamics, with the ratio exceeding 10:1 for time intervals greater than 2.5 seconds. This order-of-magnitude difference in temporal evolution provides a robust metric for wavelength discrimination in time-domain optical applications.

# Wavelength-selective encryption demonstration (630 nm and 480 nm)


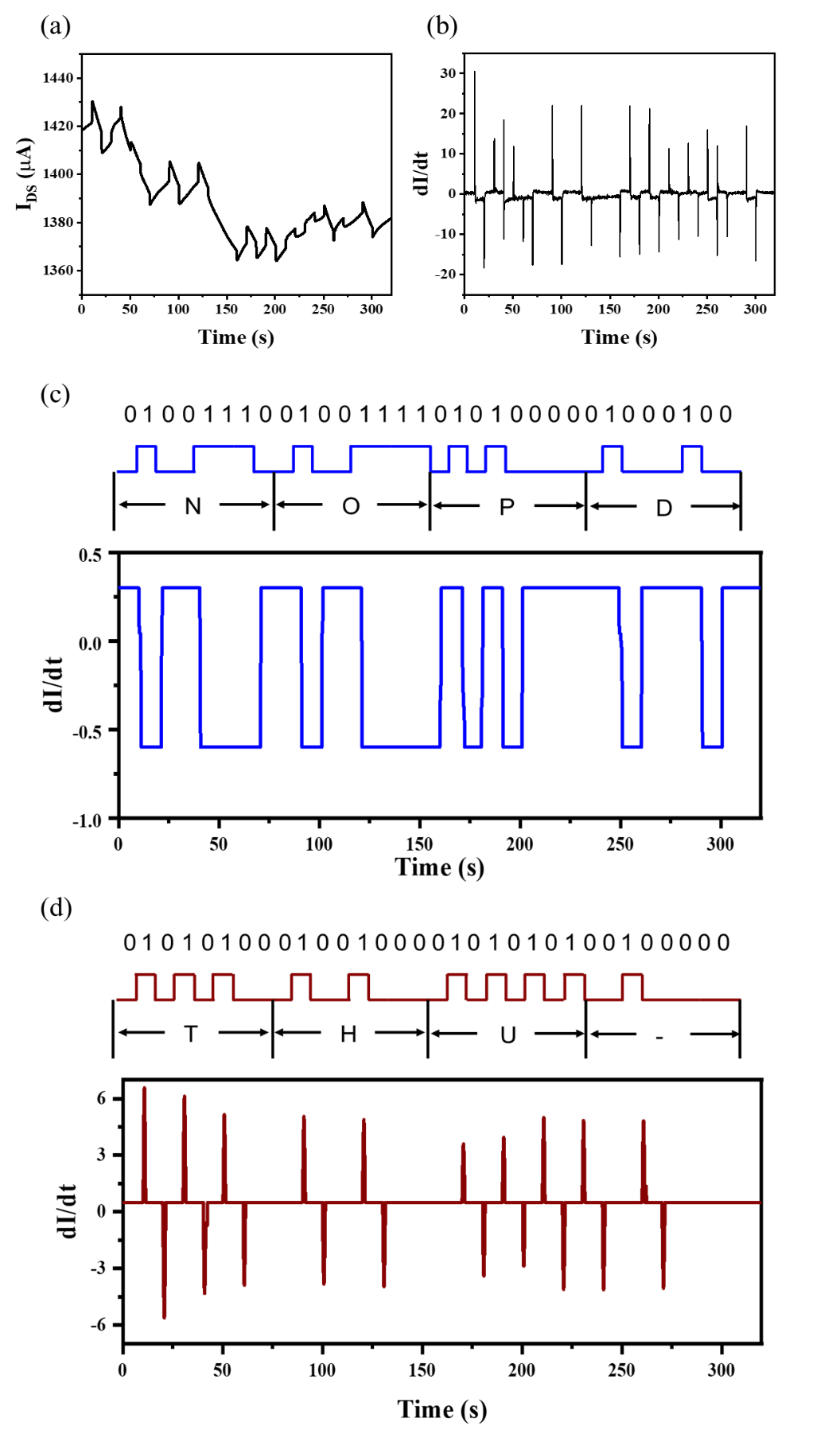


Figure S15. Encryption experiments using encrypted signals in the visible band (633 nm). (a) I-t curve of the original mixed signal. (b) the value of the I-t curve after differential operation. (c) The demultiplexed signal from 480 nm channel. (d) The demultiplexed signal from the 633 nm channel.

We demonstrate the encryption experiments using encrypted signals in the visible band (633 nm). The low absorption coefficient of visible light allows for reduced loss during underwater propagation. The original response signal is shown in Fig. S15(a), along with the corresponding dI/dt curve obtained through differential operation in Fig. S15(b). Based on distinct temporal characteristics, we successfully demultiplexed the ASCII codes ‘N’, ‘O’, ‘P’, and ‘D’ in the 480 nm channel (Fig. S15(c)), as well as ‘T’, ‘H’, ‘U’, and ‘-’ in 630 nm channel (Fig. S15(d)).

# Frequency-dependent photocurrents


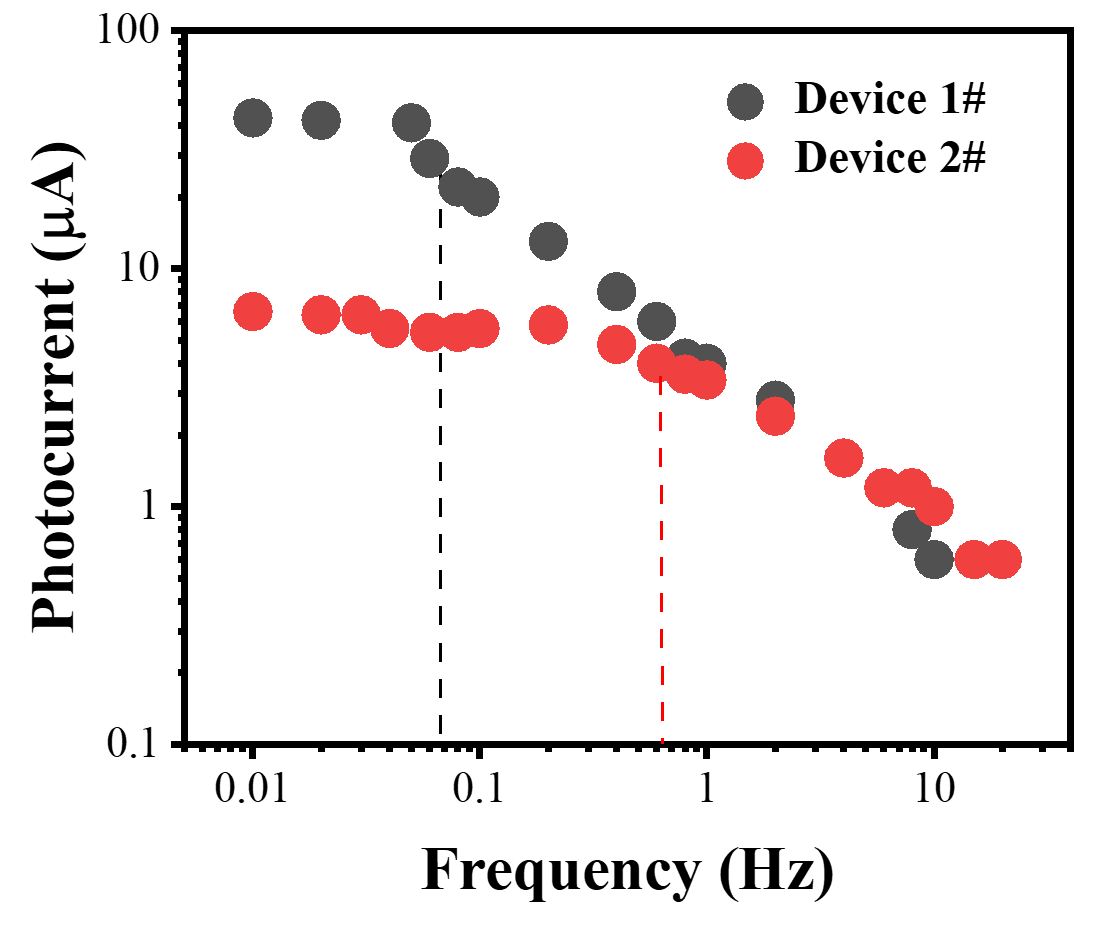


Figure S16. Frequency-dependent photocurrents in different devices.

Figure S16 presents a comparative analysis of the frequency-dependent photoresponse for two devices. Device 1# demonstrates enhanced responsivity but exhibits a limited 3 dB bandwidth of 0.07 Hz, characteristic of strong photogating effects. Conversely, Device 2# shows reduced responsivity but achieves near an order-of-magnitude improvement in bandwidth (0.6 Hz), suggesting an inverse relationship between gain and response speed in these systems.

# Performance comparison with state-of-the-art photodetectors


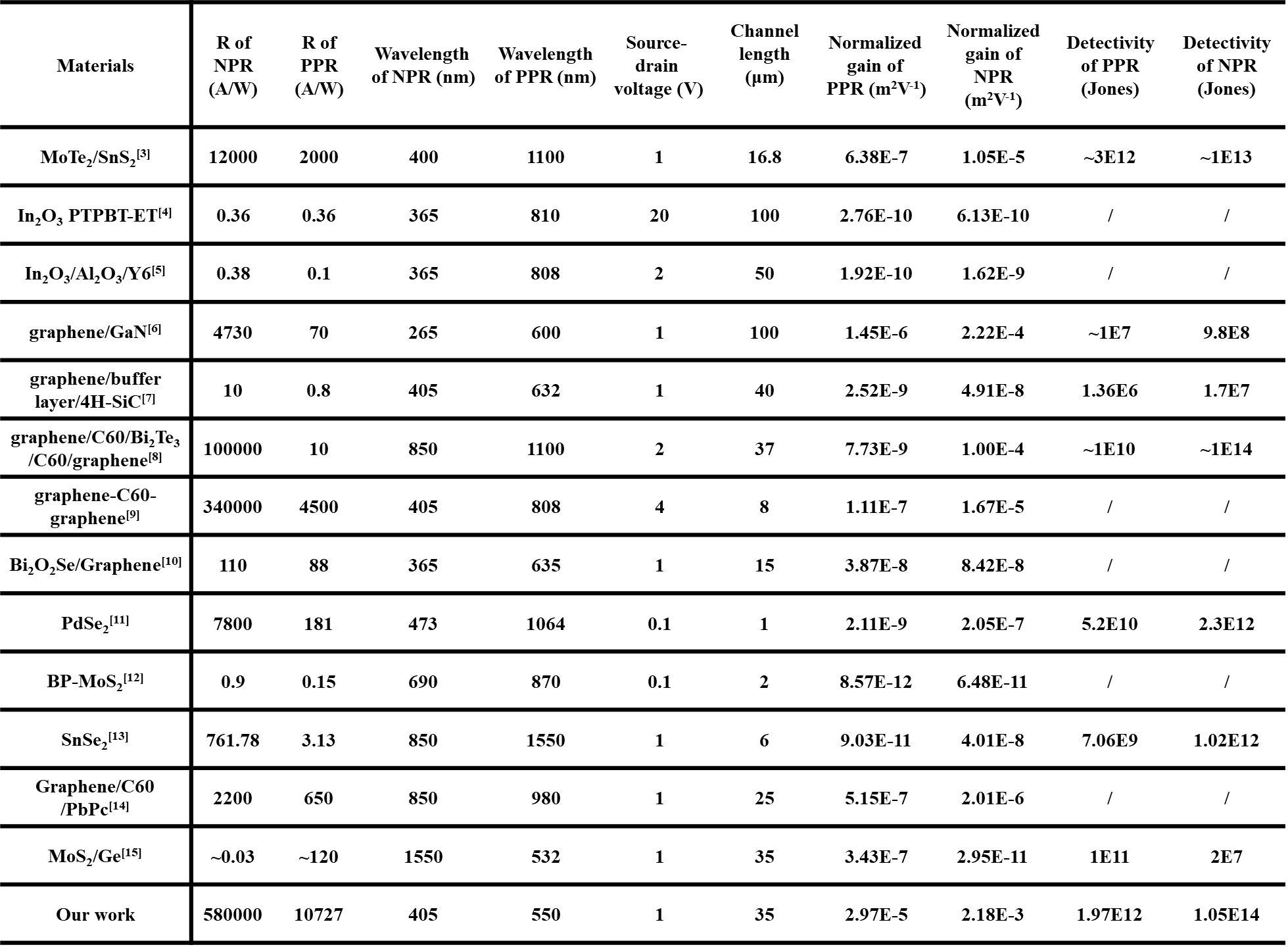


Table S1. Comparison of the relevant parameters^3-15^ in Fig. 3(d) in the main text.

# References.

1. Song, S. M.; Park, J. K.; Sul, O. J.; Cho, B. J., Determination of work function of graphene under a metal electrode and its role in contact resistance. *Nano Lett* **2012,** *12* (8), 3887-92.

2. Long, M.; Wang, P.; Fang, H.; Hu, W., Progress, Challenges, and Opportunities for 2D Material Based Photodetectors. *Advanced Functional Materials* **2018,** *29* (19).

3. Jeong, Y.; Kim, T.; Cho, H.; Ahn, J.; Hong, S.; Hwang, D. K.; Im, S., Negative Photo-Response Switching via Electron-Hole Recombination at The Type III Junction of MoTe_2_ Channel/SnS_2_ Top Layer. *Advanced Materials* **2023,** *35* (48), 2304599.

4. Li, D.; Du, J.; Chen, Y.; Wang, Y.; Tang, Y.; Liang, K.; Ren, H.; Li, F.; Song, C.; Meng, L.; Zhu, B., Schottky-Contact Hybrid Phototransistors With Bidirectional Photoresponses for Ultraviolet and Infrared Light Differentiating. *IEEE Electron Device Letters* **2022,** *43* (9), 1515-1518.

5. Li, D.; Ren, H.; Chen, Y.; Tang, Y.; Liang, K.; Wang, Y.; Li, F.; Liu, G.; Meng, L.; Zhu, B., Bidirectionally Photoresponsive Optoelectronic Transistors with Dual Photogates for All‐Optical‐Configured Neuromorphic Vision. *Advanced Functional Materials* **2023,** *33* (42), 2303198.

6. Zhang, S.; Hu, A.; Liu, Q.; Xu, L.; Ren, X.; Wang, B.; Ren, Y.; Liu, W.; Zhou, X.; Chen, S.; Guo, X., Bipolar Photoresponse in Graphene/GaN Heterostructure and its Secure Function in Free‐Space Optical Communication. *Advanced Electronic Materials* **2023,** *9* (10), 2300243.

7. Rathore, S.; Patel, D. K.; Thakur, M. K.; Haider, G.; Kalbac, M.; Kruskopf, M.; Liu, C.-I.; Rigosi, A. F.; Elmquist, R. E.; Liang, C.-T.; Hong, P.-D., Highly sensitive broadband binary photoresponse in gateless epitaxial graphene on 4H–SiC. *Carbon* **2021,** *184*, 72-81.

8. Pan, R.; Cai, Y.; Zhang, F.; Wang, S.; Chen, L.; Feng, X.; Ha, Y.; Zhang, R.; Pu, M.; Li, X.; Ma, X.; Luo, X., High Performance Graphene-C_60_ -Bismuth Telluride-C_60_-Graphene Nanometer Thin Film Phototransistor with Adjustable Positive and Negative Responses. *Advanced Science* **2023,** *10* (10), 2206997.

9. Pan, R.; Han, J.; Zhang, X.; Han, Q.; zhou, H.; Liu, X.; Gou, J.; Jiang, Y.; Wang, J., Excellent performance in vertical graphene-C_60_-graphene heterojunction phototransistors with a tunable bi-directionality. *Carbon* **2020,** *162*, 375-381.

10. Yang, C. M.; Chen, T. C.; Verma, D.; Li, L. J.; Liu, B.; Chang, W. H.; Lai, C. S., Bidirectional All‐Optical Synapses Based on a 2D Bi_2_O_2_Se/Graphene Hybrid Structure for Multifunctional Optoelectronics. *Advanced Functional Materials* **2020,** *30* (30), 2001598.

11. Jiang, J.; Xu, W.; Sun, Z.; Fu, L.; Zhang, S.; Qin, B.; Fan, T.; Li, G.; Chen, S.; Yang, S.; Ge, W.; Shen, B.; Tang, N., Wavelength-Controlled Photoconductance Polarity Switching via Harnessing Defects in Doped PdSe_2_ for Artificial Synaptic Features. *Small* **2023,** *20* (13), 2306068.

12. Jawa, H.; Varghese, A.; Ghosh, S.; Sahoo, S.; Yin, Y.; Medhekar, N. V.; Lodha, S., Wavelength‐Controlled Photocurrent Polarity Switching in BP‐MoS_2_ Heterostructure. *Advanced Functional Materials* **2022,** *32* (25), 2112696.

13. Chen, J.; Lu, S.; Hu, Y.; Yang, F.; Han, H.; Kong, L.; He, B.; Ruan, S.; Xiang, B., Ultrasensitive Bidirectional Photoresponse SnSe_2_ Photodetector Integration with Thin‐Film Lithium Niobate Photonics. *Advanced Optical Materials* **2023,** *12* (5), 2301543.

14. Dai, Q.; Hu, G.; Lv, W.; Xu, S.; Sun, L.; Schneider, G. F.; Jiang, L.; Peng, Y., Near‐Infrared Phototransistor Based on Graphene/C_60_/PbPc Heterojunction with Tunable Bidirectional Photoresponse. *Advanced Materials Interfaces* **2022,** *9* (21), 2200116.

15. You, J.; Han, Z.; Zhang, N.; Zhang, Q.; Zhang, Y.; Liu, Y.; Li, Y.; Ao, J.; Jiang, Z.; Zhong, Z.; Guo, H.; Hu, H.; Wang, L.; Zhu, Z., All-Optic Logical Operations Based on the Visible-Near Infrared Bipolar Optical Response. *Advanced Science* **2024,** *11* (40), 2404336.
